# A strategy to identify protein-N-myristoylation-dependent phosphorylation reactions of cellular proteins by using Phos-tag SDS-PAGE

**DOI:** 10.1371/journal.pone.0225510

**Published:** 2019-11-21

**Authors:** Emiko Kinoshita-Kikuta, Ayane Tanikawa, Takuro Hosokawa, Aya Kiwado, Koko Moriya, Eiji Kinoshita, Tohru Koike, Toshihiko Utsumi

**Affiliations:** 1 Department of Functional Molecular Science, Institute of Biomedical and Health Sciences, Hiroshima University, Hiroshima, Japan; 2 Graduate School of Sciences and Technology for Innovation, Yamaguchi University, Yamaguchi, Japan; 3 Department of Biological Chemistry, Faculty of Agriculture, Yamaguchi University, Yamaguchi, Japan; National Cancer Institute, UNITED STATES

## Abstract

To establish a strategy for identifying protein-N-myristoylation-dependent phosphorylation of cellular proteins, Phos-tag SDS-PAGE was performed on wild-type (WT) and nonmyristoylated mutant (G2A-mutant) FMNL2 and FMNL3, phosphorylated N-myristoylated model proteins expressed in HEK293 cells. The difference in the banding pattern in Phos-tag SDS-PAGE between the WT and G2A-mutant FMNL2 indicated the presence of N-myristoylation-dependent phosphorylation sites in FMNL2. Phos-tag SDS-PAGE of FMNL2 mutants in which the putative phosphorylation sites listed in PhosphoSitePlus (an online database of phosphorylation sites) were changed to Ala revealed that Ser-171 and Ser-1072 are N-myristoylation-dependent phosphorylation sites in FMNL2. Similar experiments with FMNL3 demonstrated that N-myristoylation-dependent phosphorylation occurs at a single Ser residue at position 174, which is a Ser residue conserved between FMNL2 and FMNL3, corresponding to Ser-171 in FMNL2. The facts that phosphorylation of Ser-1072 in FMNL2 has been shown to play a critical role in integrin β1 internalization mediated by FMNL2 and that Ser-171 in FMNL2 and Ser-174 in FMNL3 are novel putative phosphorylation sites conserved between FMNL2 and FMNL3 indicate that the strategy used in this study is a useful tool for identifying and characterizing physiologically important phosphorylation reactions occurring on N-myristoylated proteins.

## Introduction

Protein N-myristoylation is a typical lipid modification that occurs on eukaryotic and viral proteins [1–6]. In general, protein N-myristoylation is an irreversible cotranslational protein modification. In this process, myristic acid, a 14-carbon saturated fatty acid, is attached to the N-terminal Gly residue of the protein after removal of an initiating Met. In addition to cotranslational protein N-myristoylation, it has now been established that posttranslational N-myristoylation can also occur on many caspase-cleavage products, such as Bid, actin, gelsolin, or p21-activated kinase 2 (PAK2), in which proteolytic cleavage by caspase exposes an internal N-myristoylation motif [7–10]. Both cotranslational and posttranslational N-myristoylation are catalyzed by N-myristoyltransferase (NMT), which is a member of the GCN5-related N-acetyltransferase (GNAT) superfamily of proteins [11]. Many N-myristoylated proteins play critical roles in regulating cellular structure and function. These include proteins involved in a wide variety of cellular signal transduction pathways, such as protein kinases and their substrates, phosphatases, guanine nucleotide-binding proteins, E3-ubiquitin ligases, and Ca^2+^-binding proteins.

In many cases, the functions of these N-myristoylated proteins are regulated by reversible protein–membrane interactions mediated by protein N-myristoylation, as in the case of G protein α-subunits and the Src family of tyrosine kinases [1,4–6]. For example, protein N-myristoylation is required for binding of the Src family of protein kinases to cellular membranes and for intracellular signaling [1,4,6]. It has also been reported that protein kinase A (PKA) catalytic subunits released from the regulatory subunit associate with the membrane through N-myristoylation to phosphorylate membrane substrates preferentially [12]. In addition to protein kinases, protein N-myristoylation plays a critical role in membrane binding of protein kinase substrates and in their susceptibility to phosphorylation reactions. The role of protein N-myristoylation in the intracellular localization and phosphorylation of a protein kinase substrate has been well characterized in the case of myristoylated alanine-rich C kinase substrate (MARCKS), an actin cross-linking protein regulated by protein kinase C (PKC) [13,14]. Protein N-myristoylation of MARCKS was found to be involved in PKC-dependent signal transduction by targeting the soluble cytoplasmic MARCKS to the plasma membrane and then potentiating the phosphorylation of MARCKS by membrane-bound activated PKC. Thus, protein N-myristoylation plays a critical role in protein phosphorylation occurs in cells. However, no systematic strategy has been established for analyzing N-myristoylation-dependent phosphorylation of N-myristoylated proteins.

In this study, to establish a strategy for identifying protein N-myristoylation-dependent phosphorylation of cellular proteins, we performed Phos-tag SDS-PAGE analysis of wild-type (WT) and nonmyristoylated mutant (G2A-mutant) FMNL2 and FMNL3, which are phosphorylated N-myristoylated model proteins expressed in HEK293 cells [15,16]. As a result, the difference in the banding pattern in Phos-tag SDS-PAGE between the WT and G2A-mutant FMNL2 and FMNL3 revealed the presence of specific N-myristoylation-dependent phosphorylation sites in both model proteins. Phos-tag SDS-PAGE of FMNL2 mutants in which putative phosphorylation sites listed in PhosphoSitePlus (an online database of phosphorylation sites) were replaced with Ala suggested that Ser-171 and Ser-1072 are N-myristoylation-dependent phosphorylation sites in FMNL2. Similar experiments with FMNL3 demonstrated that N-myristoylation-dependent phosphorylation occurs at single Ser residue at position 174; this Ser residue is conserved between FMNL2 and FMNL3. The facts that phosphorylation of Ser-1072 in FMNL2 has been reported to play a critical role in integrin β1 internalization mediated by FMNL2 [16] and that phosphorylation of Ser-171 in FMNL2 has been proposed to increase the affinity of FMNL2 to the Rho family GTPases [17] demonstrate that the strategy used in this study is a useful tool for identifying and characterizing physiologically important phosphorylation reactions occurring on N-myristoylated proteins.

## Material and methods

### Materials

Human cDNAs coding for FMNL2 (Q96PY5-3) and FMNL3 (Q8IVF7-3) were purchased from Promega (Madison, WI, USA). ECL Prime Western Blotting Detection Reagent was sourced from GE Healthcare (Amersham, UK). The dye terminator cycle sequencing kit, Lipofectamine LTX with PLUS reagent and Hoechst 33342 were obtained from Life Technologies Corp. (Carlsbad, CA, USA). Anti-FLAG monoclonal antibody and anti-mouse IgG-FITC antibody were obtained from Sigma (St. Louis, MO, USA). Tetradec-13-ynoic acid (Alk-Myr) was sourced from Cayman Chemical Co. (Ann Arbor, MI, USA). 5-TAMRA Azide (Az-TAMRA) was purchased from Click Chemistry Tools (Scottsdale, AZ, USA). Tris(2-carboxyethyl)phosphine hydrochloride (TCEP) and tris[(1-benzyl-1*H*-1,2,3-triazol-4-yl)methyl]amine (TBTA) were obtained from Sigma (St. Louis, MO, USA). Protein G-HRP conjugate was sourced from Bio-Rad (Hercules, CA, USA). HRP-conjugated anti-mouse IgG was purchased from Cell Signaling Technology (Danvers, MA, USA). The other reagents used were obtained from Wako Pure Chemical Industries, Ltd. (Osaka, Japan) or Daiichi Pure Chemicals Co., Ltd. (Tokyo, Japan), and were of analytical or DNA grade.

### Plasmid construction

Plasmids pcDNA3-FMNL2-FLAG, pcDNA3-FMNL2-G2A-FLAG, pcDNA3-FMNL3-FLAG, and pcDNA3-FMNL3-G2A-FLAG were constructed as described previously [15]. Alanine-scanning mutants of putative phosphorylation site of FMNL2 and FMNL3 were constructed by site-directed mutagenesis using mutagenic primers (listed in S1 and S2 Tables) with pcDNA3-FMNL2-FLAG or pcDNA3-FMNL3-FLAG as template.

### Transfection of cells

Cells of HEK293 (a human embryonic kidney cell line) or COS-1 (simian virus 40-transformed African green monkey kidney cell line; American Type Culture Collection) were maintained in Dulbecco’s modified Eagle’s medium (DMEM) (Gibco BRL, Palo Alto, CA, USA) supplemented with 10% v/v fetal calf serum (FCS) (Gibco BRL). For metabolic labeling, 2 × 10^5^ cells were plated onto 35-mm-diameter dishes one day before transfection. The cells in each plate were transfected by using pcDNA3 constructs (2 μg) containing cDNAs encoding FLAG-tagged proteins, together with 2.5 μL Lipofectamine LTX and 2 μL PLUS reagent, in 1 mL of serum-free medium [18]. After incubation for 5 h at 37°C, the cells were re-fed with serum-containing medium and incubated again at 37°C for appropriate periods. For the analysis of alanine-scanning mutants of FMNL2 or FMNL3, pcDNA3 construct (0.5 μg) was transfected into HEK293 cells (1 × 10^5^) incubated in a 96-well plate. After incubation for 24 h, the transfected cells were gently washed twice with the TBS solution and immediately lysed with the sample-loading buffer for SDS-PAGE (65 mM Tris–HCl, pH 6.8 containing 1% w/v SDS, 10% v/v glycerol, 5% v/v 2-sulfanylethanol, and 0.03% w/v bromophenol blue) [19].

### Metabolic labeling of cells

The metabolic labeling of cells with myristic acid analogue (Alk-Myr) was performed as described previously [20]. HEK293 cells (2 × 10^5^) plated onto 35-mm-diameter dishes were transfected with pcDNA3 constructs (2 μg) containing cDNAs as described above and incubated at 37°C for 12 h. They were then washed once with serum-free DMEM (1 mL) and incubated for 10 h at 37°C in DMEM (1 mL) containing 2% v/v FCS and 25 μM Alk-Myr. Subsequently, the cells were washed three times with Dulbecco’s phosphate-buffered saline (DPBS), harvested, and lysed with 200 μL of RIPA buffer [50 mM Tris-HCl (pH 7.5), 150 mM NaCl, 1% v/v Nonidet P-40, 0.5% w/v sodium deoxycholate, 0.1% w/v SDS, protease inhibitors] on ice for 20 min.

### Copper(I)-catalyzed azide–alkyne cycloaddition

The cell lysates labeled with Alk-Myr (46 μL) were treated with 4 μL of freshly premixed click chemistry reaction cocktail [1 μL Az-TAMRA (5 mM), 1 μL TCEP (50 mM), 1 μL TBTA (5 mM), and 1 μL CuSO_4_·5H_2_O (50 mM)] in a total reaction volume of 50 μL for 1 h at room temperature [21]. After copper(I)-catalyzed azide–alkyne cycloaddition (CuAAC), 500 μL of MeOH was added to the sample, which was then kept at –80°C overnight. After centrifugation of the sample at 15,000 rpm at 4°C for 30 min, the supernatant was removed and the resulting pellet was washed with methanol (500 μL) and dried in air. The samples were denatured by sonication in SDS-sample buffer and subjected to SDS-PAGE. In-gel fluorescence analysis of the gel obtained by SDS-PAGE was performed by using a Typhoon FLA9500 (GE-Healthcare Bio-Sciences AB, Uppsala, Sweden).

### Western blotting

Proteins were resolved by SDS-PAGE or Phos-tag SDS-PAGE and then transferred to a PVDF membrane. The membrane was probed with an anti-FLAG antibody. Immunoreactive proteins were detected specifically by incubation with protein G-HRP conjugate or HRP-conjugated anti-mouse IgG as previously described [19,21].

### Immunofluorescence analysis and fluorescence microscopy

Immunofluorescence analysis of transfected cells was performed 24 h after transfection [22]. The cells were stained with Hoechst 33342, washed with DPBS, fixed in 4% v/v paraformaldehyde in DPBS for 15 min, and permeabilized with 0.1% v/v Triton X-100 in DPBS for 10 min at room temperature, followed by washing with 0.1% w/v gelatin in DPBS. The permeabilized cells were incubated with a specific antibody in DPBS for 1 h at room temperature. After washing with 0.1% w/v gelatin in DPBS, the cells were incubated with anti-mouse IgG-FITC antibody for 1 h at room temperature. The cells were then washed with 0.1% w/v gelatin in DPBS and observed by using a fluorescence microscope (AF7000; Leica Microsystems, Wetzlar, Germany). Fluorescence microscopic observation of cells expressing EGFP was performed 24 h after transfection without fixing cells.

### Phos-tag SDS-PAGE

Phos-tag SDS-PAGE was performed by using a 1-mm-thick, 9-cm-wide, and 9-cm-long gel on a mini-type PAGE apparatus (AE-6500; Atto Corp., Tokyo, Japan). We used a separating gel (6.3 mL) consisting of 5.5% w/v polyacrylamide and 357 mM 2-[bis(2-hydroxyethyl)amino]-2-(hydroxymethyl)propane-1,3-diol hydrochloride (Bis-Tris–HCl buffer, pH 6.8) and a stacking gel (1.8 mL) consisting of 4% w/v polyacrylamide and 357 mM Bis-Tris–HCl buffer (pH 6.8) as a neutral phosphate-affinity SDS-PAGE system [23] for phosphorylation profiling of FMNL2 and FMNL3. Phos-tag Acrylamide (20 μM) and two equivalents of ZnCl_2_ (40 μM) were added to the separating gel before polymerization. An acrylamide stock solution was prepared containing a 39:1 acrylamide–Bis mixture. The running buffer consisted of 0.10 M Tris and 0.10 M MOPS containing 0.10% w/v SDS and 5.0 mM NaHSO_3_, the latter being added immediately before use. Electrophoresis was performed at 30 mA/gel until the BPB dye reached the bottom of the separating gel. Subsequently, western blotting was performed by the wet-tank method as described previously [24].

## Results

### N-Myristoylation of both FMNL2 and FMNL3 and the induction of cellular morphological changes in an N-myristoylation-dependent manner

**Fig 1:**
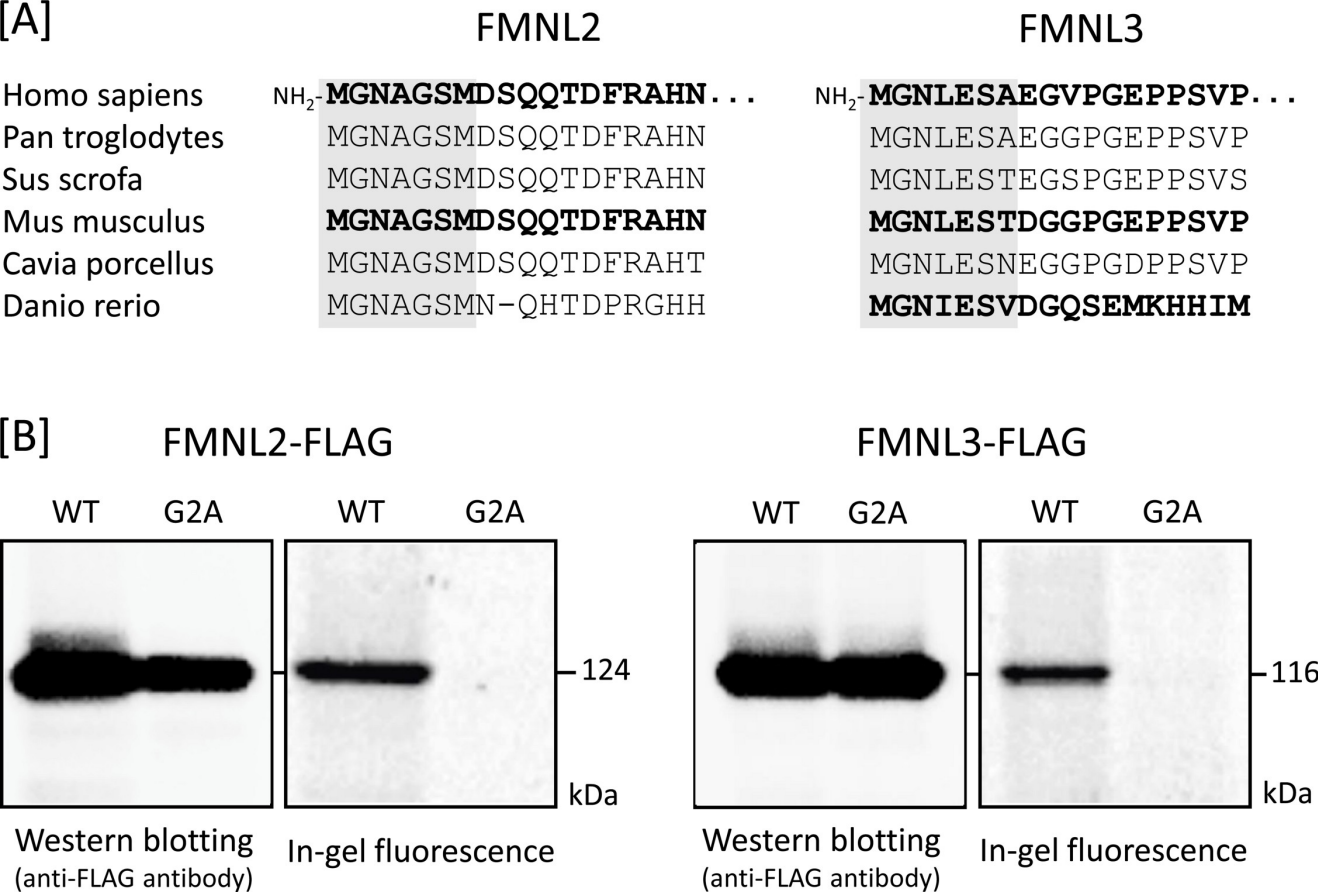
Detection of protein N-myristoylation of human FMNL2 and FMNL3 by cellular metabolic labeling. A. Interspecies alignment of the N-terminal sequences of FMNL2 and FMNL3. N-Myristoylation motifs are shown by gray highlights in the N-terminal sequences. Sequences reviewed in the UniProt database are indicated by bold-face type. B. cDNAs encoding FMNL2-FLAG, FMNL2-G2A-FLAG, FMNL3-FLAG, and FMNL3-G2A-FLAG were transfected into HEK293 cells. The expression of proteins was evaluated by western blotting analysis using an anti-FLAG antibody. Protein N-myristoylation was evaluated by metabolic labeling with a myristic acid analogue followed by click chemistry, as described in the Materials and Methods section.

**Fig 2:**
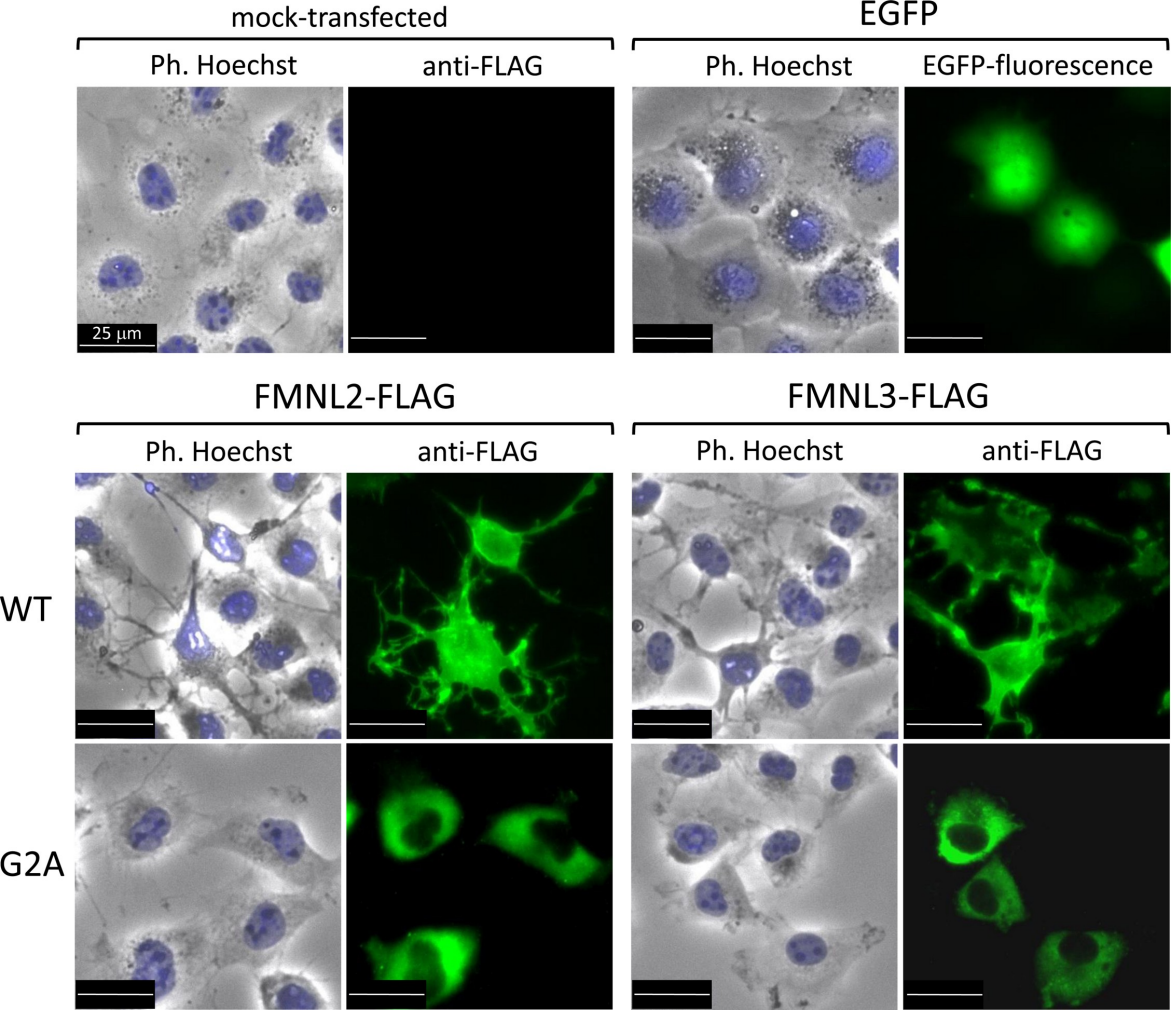
Intracellular localization of FMNL2-FLAG, FMNL2-G2A-FLAG, FMNL3-FLAG, and FMNL3-G2A-FLAG expressed in COS-1 cells. cDNAs encoding FMNL2-FLAG, FMNL2-G2A-FLAG, FMNL3-FLAG, and FMNL3-G2A-FLAG were transfected into COS-1 cells. The intracellular localization of the expressed proteins was detected by immunofluorescence staining using anti-FLAG antibody, as described in the Materials and Methods section. As control experiments, COS-1 cells transfected with empty vector (mock-transfected cells) or cDNA encoding EGFP were used. Ph.: phase contrast; Hoechst: Hoechst staining.

To confirm that protein N-myristoylation occurred on FMNL2 and FMNL3, cellular metabolic labeling experiments were performed by using cDNA encoding C-terminally FLAG-tagged FMNL2 and FMNL3. For this analysis, a nonmyristoylated G2A-mutant in which Gly-2 was replaced with Ala was used and its susceptibility to protein N-myristoylation was compared with that of WT protein. As shown in Fig 1A, interspecies alignments of the N-terminal sequence of the proteins revealed that the N-terminal N-myristoylation motif of FMNL2 and FMNL3 is highly conserved among vertebrates. The cellular metabolic labeling experiments in HEK293 cells using a bioorthogonal myristic acid analogue (Alk-Myr), followed by detection with click chemistry, revealed that both FMNL2-FLAG and FMNL3-FLAG were efficiently labeled with Alk-Myr, but that the labeling was completely inhibited on replacing Gly-2 with Ala, despite the effective expression of these proteins, as shown in Fig 1B. Immunofluorescence staining of FMNL2-FLAG and FMNL3-FLAG expressed in COS-1 cells revealed that these proteins were largely localized to the plasma membrane and that they induced remarkable cellular morphological changes such as the formation of filopodia and membrane protrusions, whereas immunofluorescence staining of FMNL2-G2A-FLAG and FMNL3-G2A-FLAG showed a diffuse cytosolic distribution without the induction of cellular morphological changes (Fig 2). As shown in Fig 2. upper panels, in un-transfected cells (mock-transfected cells) or in cells transfected with EGFP cDNA, no obvious cellular morphological change was observed. In addition, it was also revealed that cellular morphological changes induced by FMNL2-FLAG and FMNL3-FLAG were observed exclusively on transfected cells but not on un-transfected cells as shown in Fig 2, and S1 Fig. Thus, protein N-myristoylation is essential for the cellular morphological changes induced by FMNL2 and FMNL3, as previously reported [15].

### Analysis of differences in the phosphorylation status of WT and G2A-mutant FMNL2 and FMNL3 by Phos-tag SDS-PAGE

**Fig 3:**
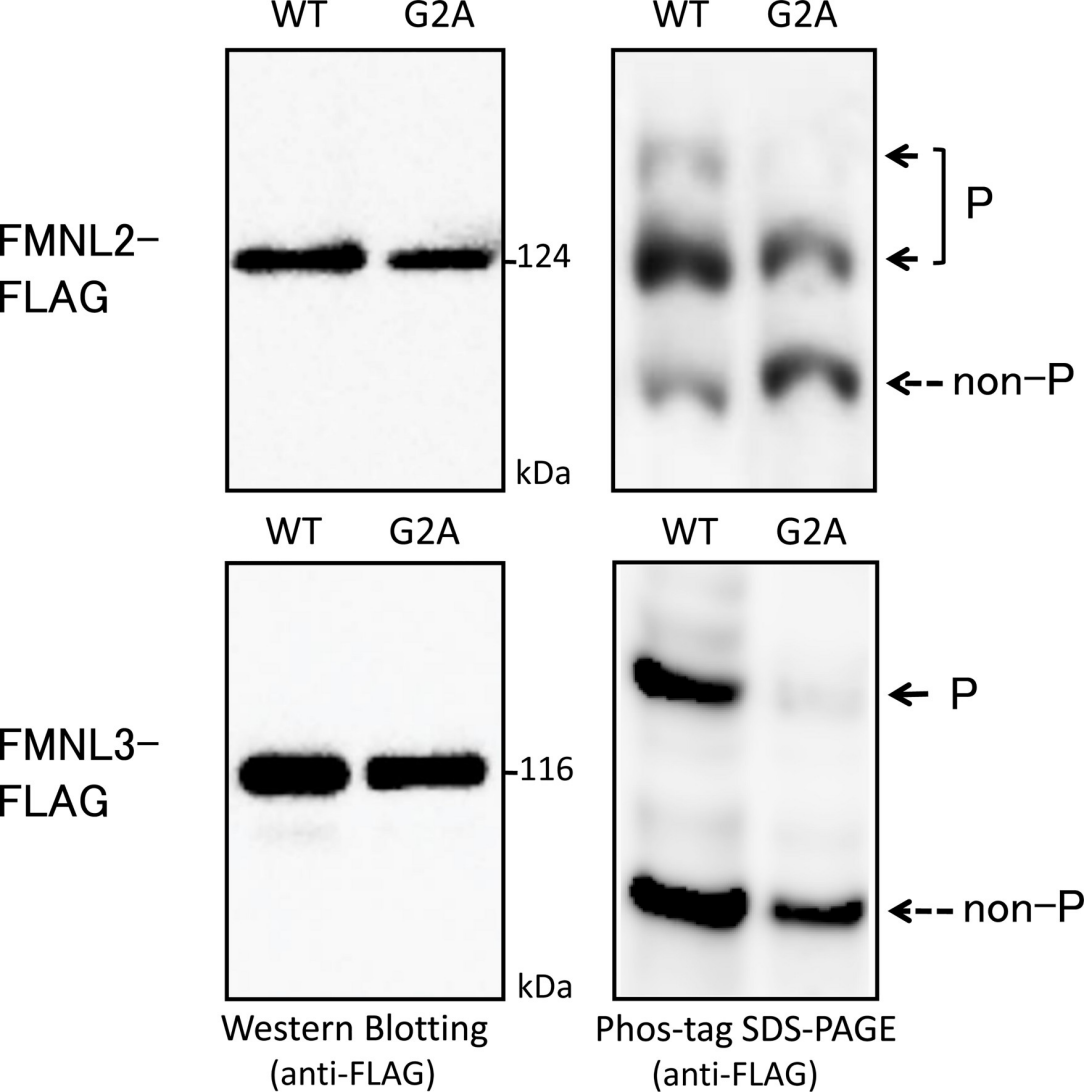
Difference in phosphorylation status between WT and G2A mutant of FMNL2 and FMNL3. Each FLAG-tagged FMNL2 (WT and G2A, upper panels) or FMNL3 (WT and G2A, lower panels) expressed in HEK293 cells was analyzed by conventional SDS-PAGE (6% w/v polyacrylamide, left panels) and by Phos-tag SDS-PAGE (20 μM Zn^2+^–Phos-tag and 5.5% w/v polyacrylamide, right panels) followed by western blotting analysis using anti-FLAG antibody. P: phosphorylated; non-P: nonphosphorylated.

We next tested the phosphorylation of FMNL2-FLAG and FMNL3-FLAG expressed in HEK293 cells. Both FMNL2-FLAG (WT and G2A, upper panels) and FMNL3-FLAG (WT and G2A, lower panels) expressed in HEK293 cells were analyzed by SDS-PAGE followed by western blotting analysis using anti-FLAG antibody (Fig 3). In the conventional SDS-PAGE, no difference was observed in the banding pattern between the WT and G2A-mutant proteins in FMNL2 (124 kDa, upper-left panel) or FMNL3 (116 kDa, lower-left panel). On the other hand, Phos-tag SDS-PAGE showed the presence of multiple migration bands in both WT proteins of FMNL2 and FMNL3. Furthermore, there were significant differences in the banding patterns between the WT proteins and the G2A mutants (right panels). In WT FMNL2, three bands were visualized. Interestingly, the top band disappeared and the bottom band became stronger in G2A-mutant FMNL2. We assigned the upper and middle bands to phosphorylated (P) species, and the lower band to a nonphosphorylated (non-P) species. For WT FMNL3, two bands were visualized and we assigned the upper and lower bands to phosphorylated (P) and nonphosphorylated (non-P) species, respectively. The difference in the banding pattern between WT and G2A-mutant FMNL2 and FMNL3 strongly indicated that N-myristoylation-dependent phosphorylation occurred in these proteins.

### Identification of phosphorylated residues in FMNL2 and FMNL3 by alanine-scanning mutagenesis

**Fig 4:**
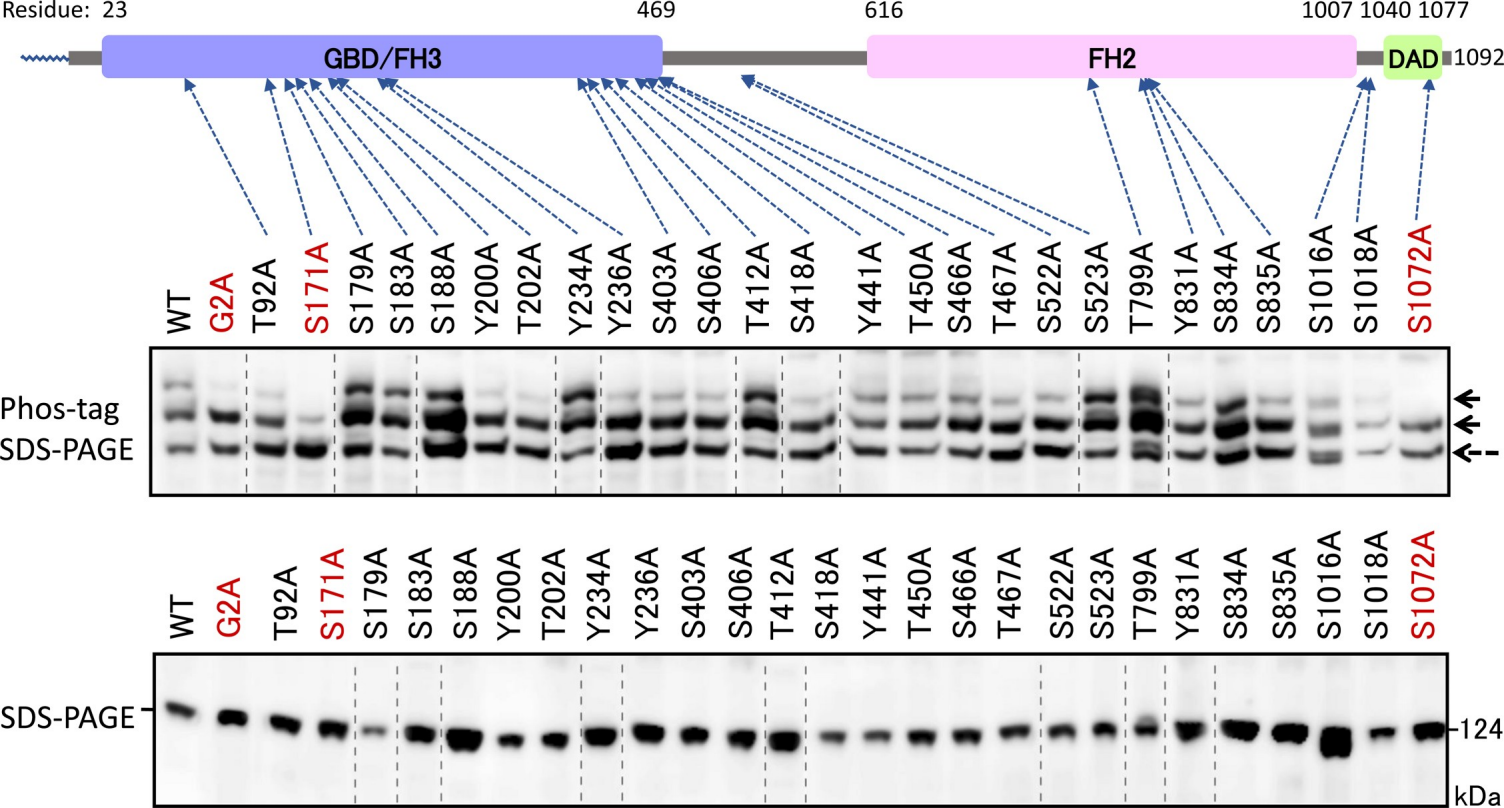
Identification of phosphorylated residues in FMNL2 by alanine-scanning mutagenesis. FLAG-tagged FMNL2 (WT) and its mutants (Ala-scanning mutants) expressed in HEK293 cells were analyzed by conventional SDS-PAGE (6% w/v polyacrylamide, lower panel) and by Phos-tag SDS-PAGE (20 μM Zn^2+^–Phos-tag and 5.5% w/v polyacrylamide, upper panel) followed by western blotting analysis using anti-FLAG antibody. The gel image in the raw image data (S2 Fig.) was spliced to arrange the lanes appropriately. The spliced position was indicated by vertical dashed line.

**Fig 5:**
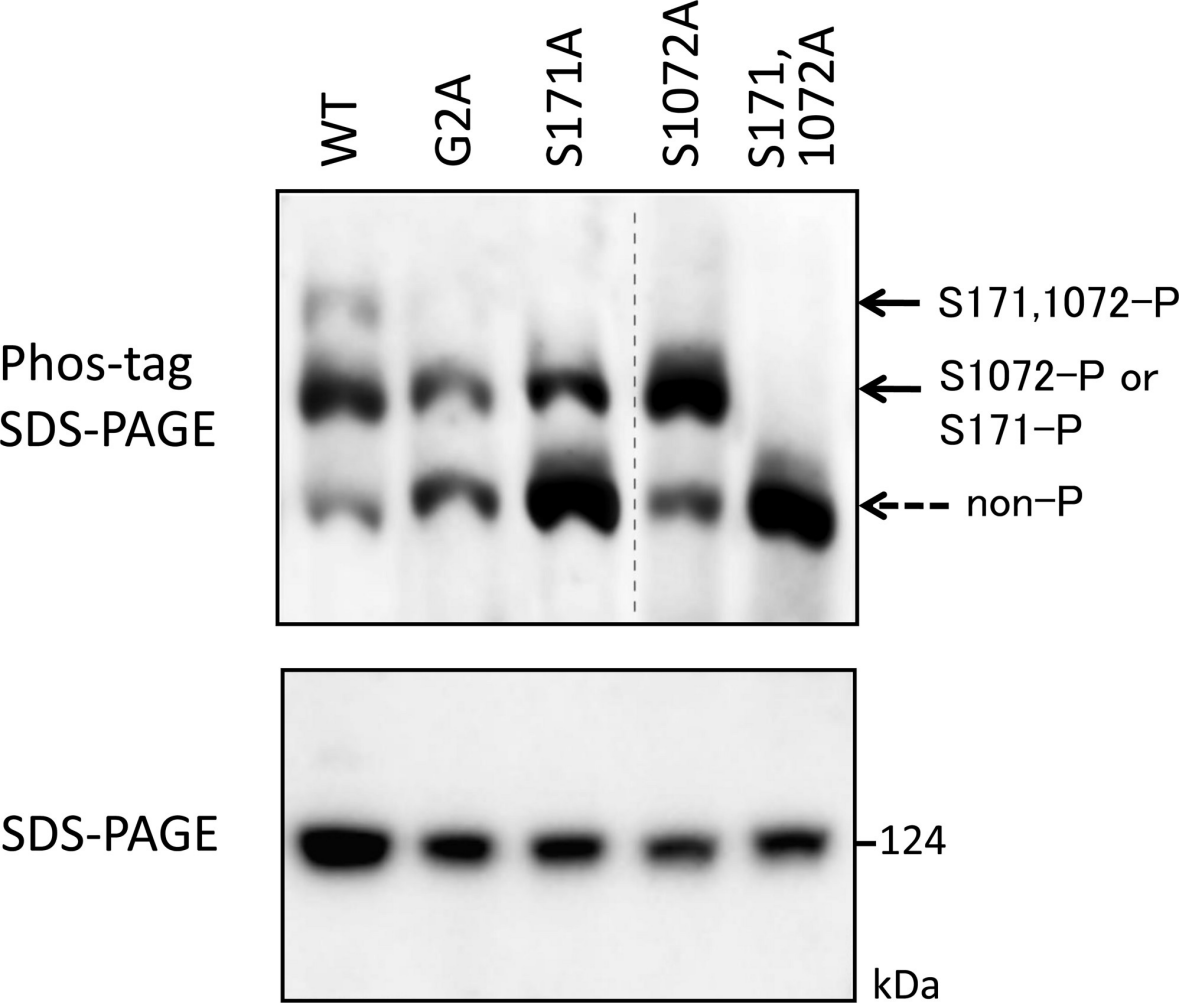
Assignment of phosphorylated species corresponding to up-shifted bands of FMNL2. FLAG-tagged FMNL2 (WT) and its mutants (G2A, S171A, S1072A, and S171A, S1072A) expressed in HEK293 cells were analyzed by conventional SDS-PAGE (6% w/v polyacrylamide, lower panel) and by Phos-tag SDS-PAGE (20 μM Zn^2+^–Phos-tag and 5.5% w/v polyacrylamide, upper panel) followed by western blotting analysis using anti-FLAG antibody. The phosphorylated species corresponding to each up-shifted band was assigned as shown by the respective solid arrows (S171,1072-P, S1072-P or S171-P). The bottom band corresponds to the nonphosphorylated species (non-P). The gel image in the raw image data (S2 Fig.) was spliced to arrange the lanes appropriately. The spliced position was indicated by vertical dashed line.

To identify the phosphorylation sites of FMNL2, we used the information listed in PhosphoSitePlus (http://www.phosphosite.org/homeAction), which is an online database provided by Cell Signaling Technology, Inc. (Danvers, MA, USA) that lists the locations of phosphorylation sites, as described previously [19]. Site-directed mutagenesis was performed by substitutions of the 26 putative phosphorylation sites of FMNL2 with Ala by using a FLAG-tagged FMNL2 (WT) cDNA. We initially attempted to use Phos-tag SDS-PAGE to detect the protein phosphorylation occurring on the WT protein, the G2A-mutant, and the 26 Ala-substituted mutants of the protein, transiently expressed in HEK 293 cells under the same culture conditions. Subsequent western blotting analysis with anti-FLAG antibody revealed that the top band disappeared in the two Ala-substituted mutants S171A and S1072A in the same manner as in the G2A mutant **(**Fig 4). These results indicated that the two serine residues are constitutive phosphorylation sites responsible for the up-shifted bands in the WT protein. We next used Phos-tag SDS-PAGE to analyze one additional double mutant of FMNL2 constructed by substitutions of both Ser-171 and Ser-1072 residues of WT FMNL2 with Ala residues (Fig 5). The two phosphorylated species corresponding to the top and middle bands (see Fig 5) completely disappeared in the double mutant. Thus, the top band in the WT protein was taken to correspond to the diphosphorylated species modified at Ser-171 and Ser-1072, and middle band was taken to correspond to the monophosphorylated species modified at Ser-171 or Ser-1072, as shown by the corresponding solid arrows in the upper panel of Fig 5. In this experiment, we noted that each of two monophosphorylated species modified at Ser-171 or Ser-1072 migrated to the same position in the Phos-tag SDS-PAGE gel under the experimental conditions. On comparing the banding patterns in the Phos-tag SDS-PAGE of the S171A and S1072A mutants, a much stronger nonphosphorylated (non-P) band was observed in the S171A mutant than in S1072A mutant, indicating that much more efficient phosphorylation occurred on Ser-171 than on Ser-1072 in WT FMNL2. The fact that some extent of monophosphorylated protein band was observed on nonmyristoylated G2A-mutant of FMNL2 suggested that a portion of Ser-171 or Ser-1072 in FMNL2 was phosphorylated independently to the protein N-myristoylation.

**Fig 6:**
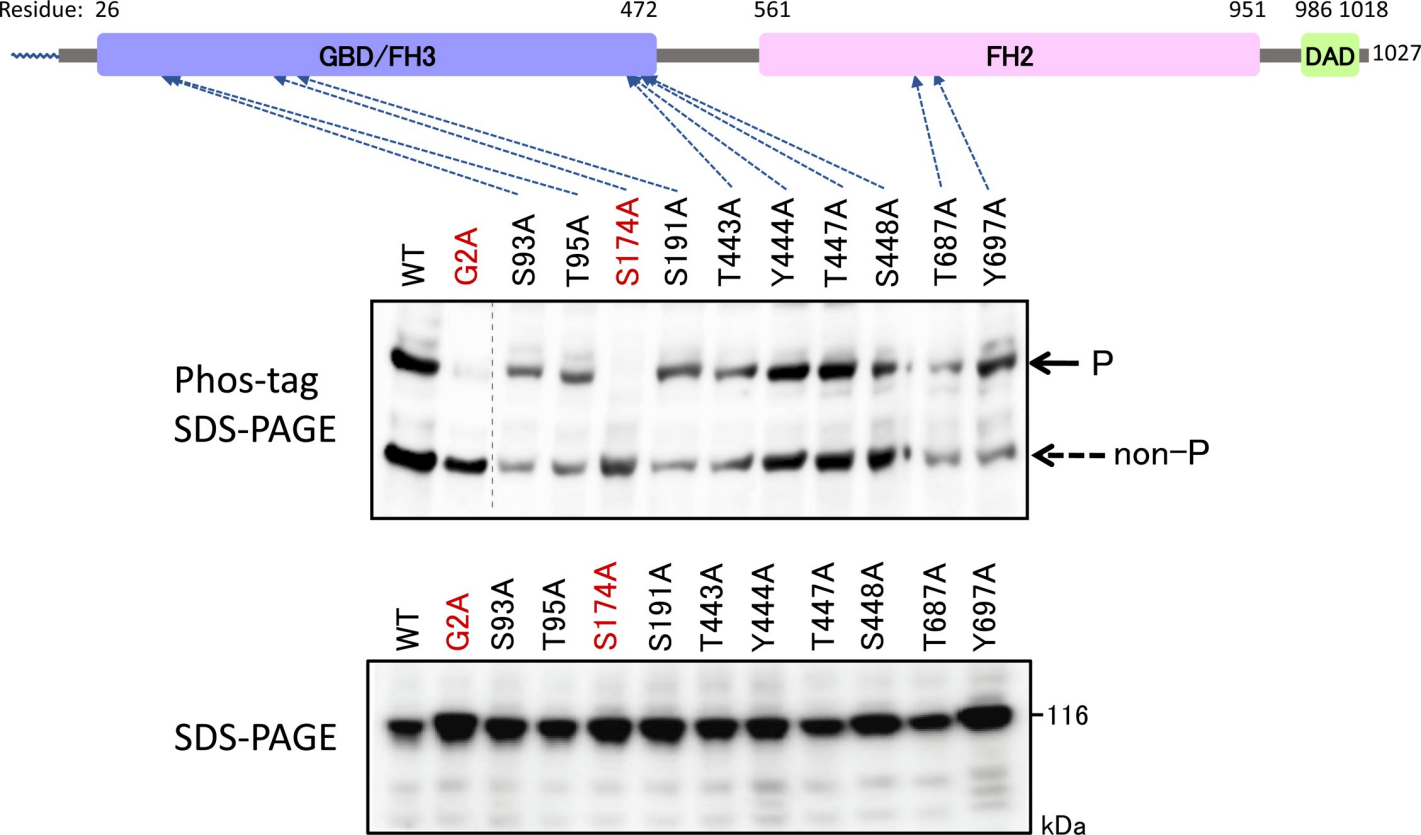
Identification of phosphorylated residues in FMNL3 by alanine-scanning mutagenesis. FLAG-tagged FMNL3 (WT) and its mutants (Ala-scanning mutants) expressed in HEK293 cells were analyzed by conventional SDS-PAGE (6% w/v polyacrylamide, lower panel) and by Phos-tag SDS-PAGE (20 μM Zn^2+^–Phos-tag and 5.5% w/v polyacrylamide, upper panel) followed by western blotting analysis using anti-FLAG antibody. The upper band was assigned to the phosphorylated species, as shown by the solid arrow (P), and the lower band was assigned to the nonphosphorylated species (non-P). The gel image in the raw image data (S2 Fig.) was spliced to arrange the lanes appropriately. The spliced position was indicated by vertical dashed line.

In the same way, alanine-scanning mutagenesis was carried out by using a FLAG-tagged FMNL3 (WT) cDNA. Phos-tag SDS-PAGE followed by western blotting analysis using anti-FLAG antibody revealed that the phosphorylated species corresponding to the upper band (P) disappeared in the S174A mutant in the same manner as in the G2A mutant, as shown in Fig 6, upper panel. The upper band was assigned to a phosphorylated species modified at Ser-174, shown by a solid arrow in the upper panel. These results therefore showed that N-myristoylation-dependent phosphorylation occurs at the Ser174 residue in FMNL3.

### Possible physiological role of the protein-N-myristoylation-dependent phosphorylation reactions of FMNL2 and FMNL3

**Fig 7:**
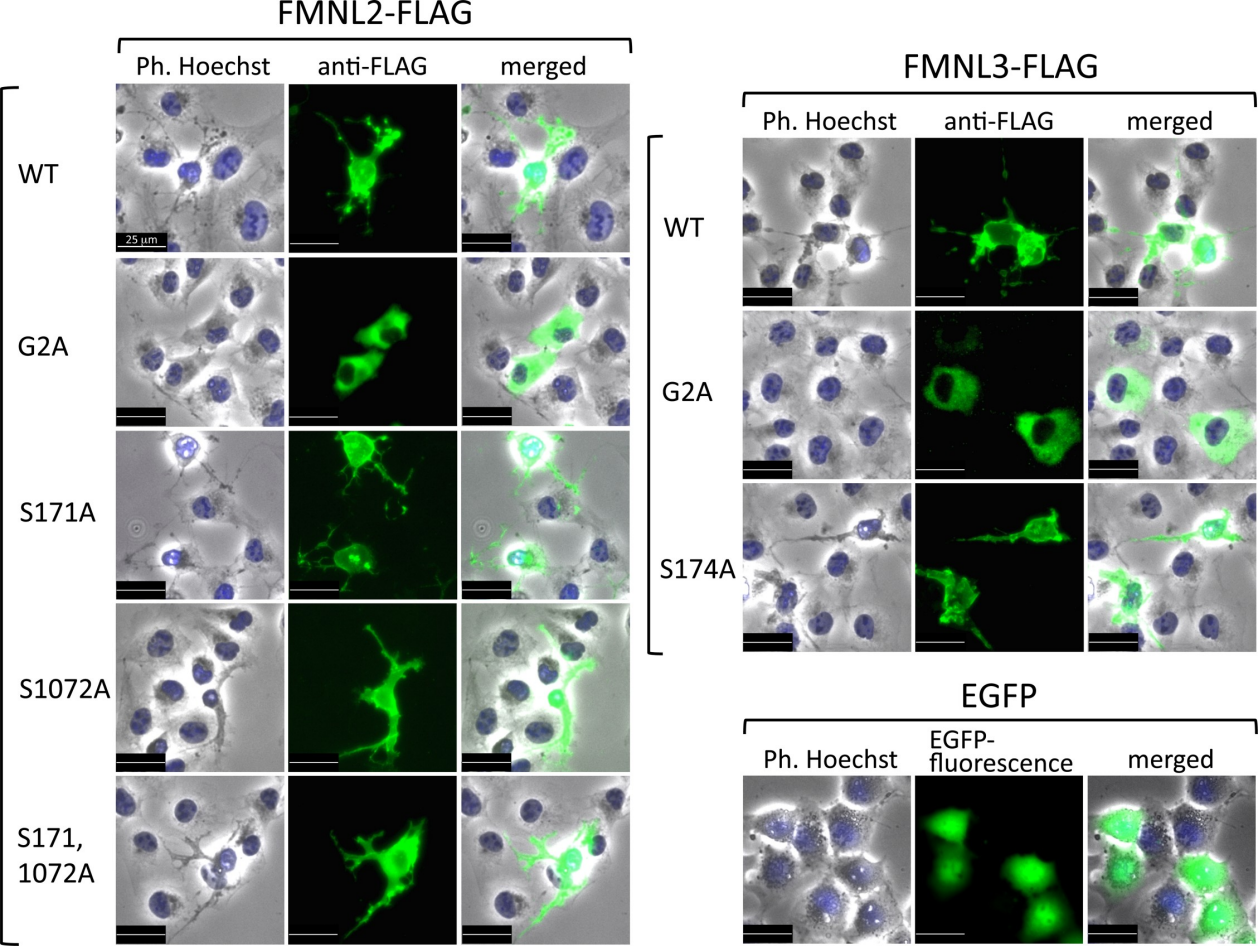
Phosphorylation of Ser171 in FMNL2 or Ser174 in FMNL3 is not involved in the cellular morphological changes induced by FMNL2 and FMNL3. To determine the possible physiological role of protein phosphorylation occurring on FMNL2 and FMNL3, the effects of overexpression of phosphorylation-deficient mutants of FMNL2 and FMNL3 on cellular morphology were evaluated. cDNA encoding FLAG-tagged WT, G2A, and phosphorylation-deficient mutants of FMNL2 and FMNL3 were transfected into COS-1 cells, and immunofluorescence analysis was performed as described in the Materials and Methods section. As a control, COS-1 cells transfected with cDNA encoding EGFP were used.

Phosphorylation of Ser1072 in FMNL2 has been shown to play a critical role in stimuli-induced integrin β1 internalization mediated by FMNL2 [16]. Therefore, it is quite reasonable that Ser1072 in FMNL2 was identified as the N-myristoylation-dependent phosphorylation site. However, phosphorylation of Ser171 in FMNL2 or Ser174 in FMNL3 has not been reported previously. To determine the possible physiological role of protein phosphorylation occurring on these two conserved Ser residues, we evaluated the effect of overexpression of phosphorylation-deficient mutants of FMNL2 and FMNL3 on cellular morphological changes. As shown in Fig 7, WT FMNL2-FLAG and FMNL3-FLAG induced significant cellular morphological changes, whereas no cellular morphological changes were observed with the corresponding nonmyristoylated G2A-mutants. In the case of phosphorylation-deficient mutants of FMNL2 (S171A-FMNL2-FLAG, S1072A-FMNL2-FLAG, or S171,1072A-FMNL2-FLAG) and FMNL3 (S174A-FMNL3-FLAG), significant morphological changes, similar to those induced by WT FMNL2 and FMNL3, were observed. It is therefore probable that the phosphorylation reactions detected in the present study are not directly involved in the cellular morphological changes induced by FMNL2 and FMNL3. Thus, the physiological role of protein phosphorylation at Ser171 in FMNL2 or Ser174 in FMNL3 remains to be elucidated.

### Conservation of phosphorylation sites found in FMNL2 and FMNL3

**Fig 8:**
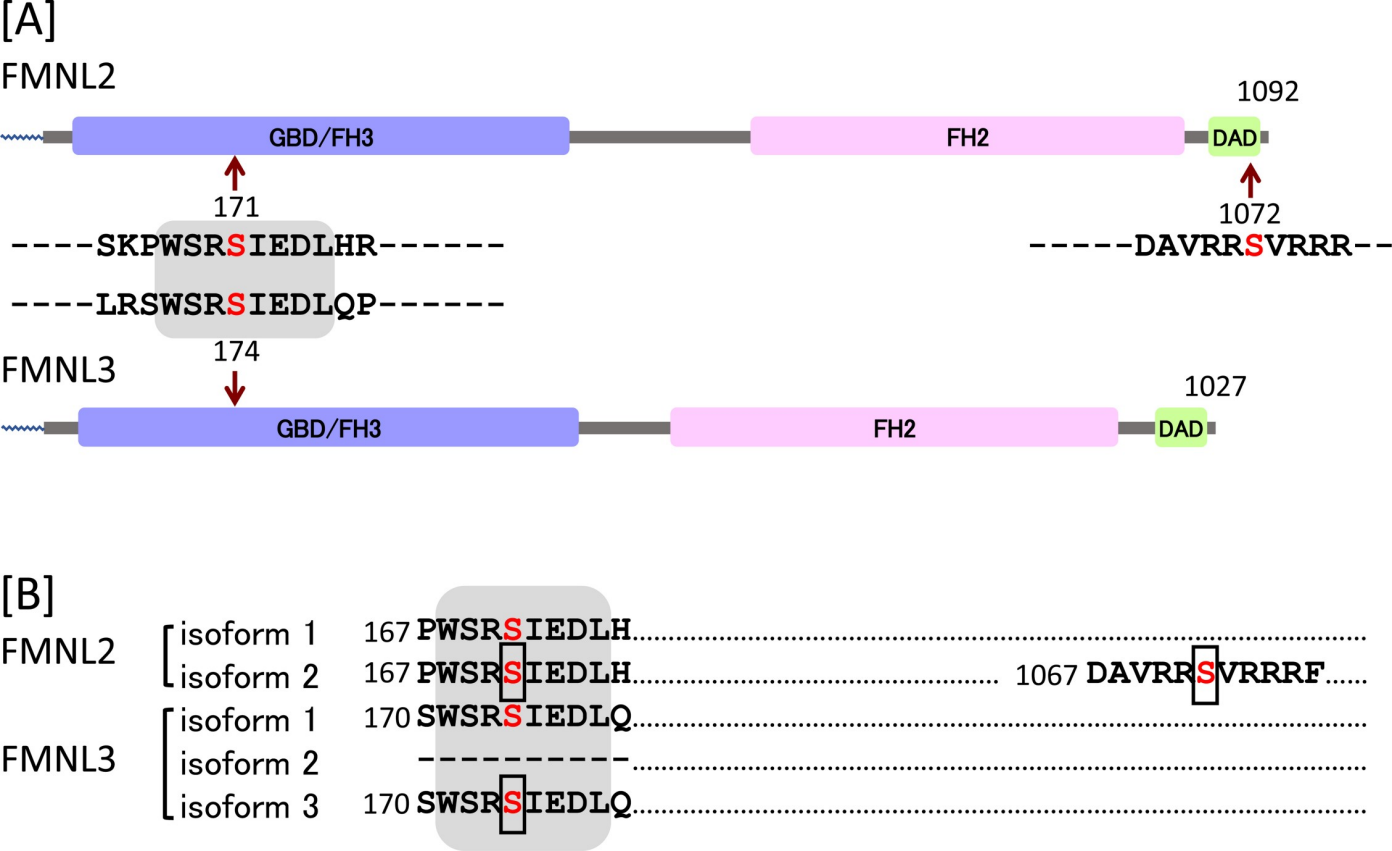
Conservation of phosphorylation sites found in FMNL2 and FMNL3. A. Amino acid sequence around the phosphorylation sites found in FMNL2 and FMNL3 were compared. The gray highlight indicates a conserved amino acid sequence. Red characters indicate the phosphorylation sites detected in this study. B. Conservation of phosphorylation sites among isoforms of FMNL2 and FMNL3. The gray highlight indicates the conserved amino acid sequence. The boxes indicate the phosphorylation sites detected in this study.

When amino acid sequences around the N-myristoylation-dependent phosphorylation sites identified in FMNL2 and FMNL3 were compared, it was found that the amino acid sequence surrounding the phosphorylation sites in the N-terminal GBD/FH3 domain were highly conserved between FMNL2 and FMNL3. In fact, the sequences of eight amino acids around the phosphorylated Ser residue (NH_2_–Trp–Ser–Arg–Ser(P)–Ile–Glu–Asp–Leu–COOH) were exactly the same, as shown in Fig 8A. In addition, this amino acid sequence was conserved among most of the isoforms of FMNL2 and FMNL3 (Fig 8B). In contrast, the phosphorylation site found in the C-terminal DAD domain of FMNL2 was not conserved between FMNL2 and FMNL3, and was specifically observed only in isoform 2 of FMNL2.

## Discussion

Since the discovery of protein N-myristoylation in the catalytic subunit of protein kinase A (PKA), many proteins in various signal transduction pathways have been shown to be N-myristoylated [1–6,12–14]. In particular, many protein kinases and protein kinase substrates have been found to be N-myristoylated, and it has been shown that specific protein–lipid or protein–protein interactions mediated by protein N-myristoylation play critical roles in the physiological functions of phosphorylation reactions performed by these proteins. Mass spectrometry (MS)-based shotgun phosphoproteomics has emerged as the main technique for the discovery and characterization of phosphoproteins. However, no systematic strategy has been developed for identifying and characterizing N-myristoylation-dependent phosphorylation of a given N-myristoylated protein. In this study, Phos-tag SDS-PAGE was used to establish a strategy for identifying and characterizing protein N-myristoylation-dependent phosphorylation of cellular proteins. This electrophoretic technique was developed to detect changes in the mobilities of phosphoprotein species in comparison with those of their nonphosphorylated counterparts. Phos-tag SDS-PAGE is capable of separating multiple phosphoprotein species that contain different numbers of phosphoryl groups or identical numbers of phosphoryl groups attached at different locations within a given protein molecule [25–27].

In this study, FMNL2 and FMNL3, members of the formin family of proteins, were used as models for phosphorylated N-myristoylated proteins. Formin family proteins have been found to promote the formation of actin networks and to be involved in the regulation of cellular actin-dependent processes such as cell adhesion, migration, division, morphogenesis, or intracellular trafficking of protein [28–30]. Formins are defined by a highly conserved C-terminal formin homology 2 (FH2) domain that mediates actin polymerization [31,32]. The FH2 domain is preceded by a formin homology 1 (FH1) domain that binds to profilin [33]. Most formins function as effectors for Rho-GTPases and are regulated by intramolecular binding between the diaphanous inhibitory (DID) and autoregulatory (DAD) domains [34]. A subset of formins is classified as diaphanous-related formins (DRFs), based on the presence of a C-terminal DAD domain. FMNL2 and FMNL3 are members of formin-like proteins, a family of DRFs in mammals. A database search of UniProt Knowledgebase (UniProtKB; https://www.uniprot.org) revealed that FMNL2 and FMNL3 have two and three isoforms, respectively. In these isoforms, the N-myristoylation motif at the N-terminus is highly conserved and differences in amino acid sequence are observed mainly in about 30 amino acids in the C-terminus. Since isoform 2 of FMNL2 and isoform 3 of FMNL3 have been used in most studies on FMNL2 and FMNL3, we used these isoforms in the present study.

With respect to protein N-myristoylation, we have demonstrated that FMNL2 and FMNL3 are N-myristoylated and that protein N-myristoylation is required for plasma membrane localization and for cellular morphological changes induced by overexpression of FMNL2 and FMNL3 [15,35]. With regard to protein phosphorylation of FMNL2 and FMNL3, it has been shown that phosphorylation of FMNL2 by PKC plays a critical role in the physiological function of FMNL2 [16]. FMNL2 is unique among formins in that it is upregulated in several metastatic cancers and is involved in the behavior and progression of cancer cells [36–38]. It has been reported that PKCα associates with and phosphorylates FMNL2 at Ser-1072 within its DAD domain, leading to the release of formin autoinhibition. Phosphorylation of FMNL2 triggers its rapid relocation and promotes its interaction with the cytoplasmic tails of the α-integrin subunits for β1-integrin endocytosis [16]. Thus, FMNL2 drives β1-integrin internalization and invasive motility of cancer cells in a phosphorylation-dependent manner. In these mechanisms, plasma membrane binding of FMNL2 mediated by protein N-myristoylation seems to play vital role in the recognition of FMNL2 by the membrane-bound activated form of PKCα. In fact, many studies have shown that N-myristoylation-dependent membrane binding plays critical role in the phosphorylation of substrate proteins by PKC [13,14,39,40].

When Phos-tag SDS-PAGE was performed on WT and nonmyristoylated-mutant (G2A-mutant) FMNL2 and FMNL3, significant differences in the banding patterns of WT and G2A-mutant FMNL2 and FMNL3 were observed (Fig 3), indicating the presence of specific N-myristoylation-dependent phosphorylation sites in FMNL2 and FMNL3. In this case, however, the difference in the banding pattern between WT protein and G2A mutant of FMNL2 and FMNL3 was not similar to each other, suggesting that protein N-myristoylation has different effects on the phosphorylation reactions of FMNL2 and FMNL3.

We next tried to identify the N-myristoylation-dependent phosphorylation sites in FMNL2 and FMNL3 by using our recently established Phos-tag-based strategy for identifying specific phosphorylation sites from existing information on the location of phosphorylation sites (phosphoproteomic data) deposited in the database PhosphoSitePlus (http://www.phosphosite.org) [19]. Phos-tag SDS-PAGE of FMNL2 mutants in which putative phosphorylation sites listed in PhosphoSitePlus were changed to Ala suggested that Ser-171 and Ser-1072 are N-myristoylation-dependent phosphorylation sites in FMNL2. Similar experiments on FMNL3 demonstrated that N-myristoylation-dependent phosphorylation occurs at a single Ser residue at position 174, a Ser residue that is conserved between FMNL2 and FMNL3.

As previously described, phosphorylation of Ser-1072 in FMNL2 by PKCα has been reported to play critical role in integrin β1 internalization mediated by FMNL2 [16]. The amino acid sequence RRSVR, including Ser-1072, found in the C-terminal DID domain constitutes a consensus motif for classical PKCs [41]. This sequence is not conserved among FMNL2 and FMNL3, and is found only in isoform 2 of FMNL2 (Fig 8). These observations are consistent with the experimental results obtained in the present study that phosphorylation of the PKC consensus motif located at the C-terminal DID domain was observed for FMNL2 only, and was not observed for FMNL3.

Regarding the conserved N-terminal phosphorylation sites of FMNL2 and FMNL3, no direct experimental results indicating that these amino acids undergo phosphorylation has been reported. However, proteomic analyses have identified a phosphorylation site for Ser-171 in FMNL2 [42]. In addition, a study using the S171DD mutant, in which two aspartates were introduced at the site of Ser-171 as a mimic of phosphorylated Ser-171, showed that phosphorylation of Ser-171 in FMNL2 might increase its affinity toward cdc42, a Rho family GTPase [17]. It is therefore probable that phosphorylation of Ser-171 in FMNL2 or of Ser-174 in FMNL3 positively affects the binding of these formins to the Rho family of GTPases. As shown in Fig 7, analyses of the effects of phosphorylation-deficient mutants of FMNL2 and FMNL3 on cellular morphology suggested that the phosphorylation reaction detected in the present study does not significantly affect cellular morphological changes induced by FMNL2 and FMNL3. Thus, the physiological role of protein phosphorylation at Ser-171 in FMNL2 or at Ser-174 in FMNL3 remains to be elucidated.

In the present study, we have shown that an electrophoretic analysis by Phos-tag SDS-PAGE of the WT protein and its nonmyristoylated G2A-mutant, expressed in mammalian cells, is an efficient strategy for identifying and characterizing the physiologically important phosphorylation reaction of the N-myristoylated protein. The advantages of the use of this strategy are, first, the use of MS is not required; secondly, specific phosphorylation sites can be determined by utilizing phosphoproteomic data; and, thirdly, the cellular physiological role of protein phosphorylation at a distinct position can be studied by using phosphorylation-deficient mutants. To confirm the efficacy of this strategy in studies on the N-myristoylation-dependent phosphorylation reaction, we are currently studying the phosphorylation reactions of many N-myristoylated kinases and kinase substrates.

## Supporting information

S1 Table PCR primers for FMNL2 mutagenesis. (DOCX)

S2 Table PCR primers for FMNL3 mutagenesis. (DOCX)

S1 Fig FMNL2 and FMNL3 expressed in COS-1 cells induced cellular morphological changes in an N-myristoylation dependent manner. The merged images of phase contrast images and fluorescence microscopic images of COS-1 cells transfected with cDNA coding for wild type and G2A-mutants of FMNL2-FLAG and FMNL3-FLAG presented in Fig 2 were shown to demonstrate that induction of cellular morphological changes by FMNL2 and FMNL3 is dependent on protein N-myristoylation.
(TIF)

S2 Fig Raw images of blot/gel data shown in Figs 1, 3, 4, 5 and 6. (TIF)

## References

[1] Udenwobele DI, Su RC, Good SV, Ball TB, Varma Shrivastav S, Shrivastav A. Myristoylation: An important protein modification in the immune response. Front Immunol. 2017;8:751 10.3389/fimmu.2017.00751 28713376 PMC5492501

[2] Peng T, Thinon E, Hang HC. Proteomic analysis of fatty-acylated proteins. Curr Opin Chem Biol. 2016;30:77–86. 10.1016/j.cbpa.2015.11.008 26656971 PMC4731282

[3] Giglione C, Fieulaine S, Meinnel T. N-terminal protein modifications: Bringing back into play the ribosome. Biochimie. 2015;114:134–146. 10.1016/j.biochi.2014.11.008 25450248

[4] Resh MD. Trafficking and signaling by fatty-acylated and prenylated proteins. Nat Chem Biol. 2006;2:584–590. 10.1038/nchembio834 17051234

[5] Farazi TA, Waksman G, Gordon JI. The biology and enzymology of protein N-myristoylation. J Biol Chem. 2001;276:39501–39504. 10.1074/jbc.R100042200 11527981

[6] Resh MD. Fatty acylation of proteins: new insights into membrane targeting of myristoylated and palmitoylated proteins. Biochim Biophys Acta. 1999;1451:1–16. 10.1016/s0167-4889(99)00075-0 10446384

[7] Zha J, Weiler S, Oh K-J, Wei MC, Korsmeyer SJ. Posttranslational N-myristoylation of BID as a molecular switch for targeting mitochondria and apoptosis. Science. 2000;290:1761–1765. 10.1126/science.290.5497.1761 11099414

[8] Utsumi T, Sakurai N, Nakano K, Ishisaka R. C-Terminal 15 kDa fragment of cytoskeletal actin is posttranslationally N-myristoylated upon caspase-mediated cleavage and targeted to mitochondria. FEBS Lett. 2003;539:37–44. 10.1016/s0014-5793(03)00180-7 12650923

[9] Sakurai N, Utsumi T. Posttranslational N-myristoylation is required for the anti-apoptotic activity of human tGelsolin, the C-terminal caspase cleavage product of human gelsolin. J Biol Chem. 2006;281:14288–14295. 10.1074/jbc.M510338200 16556605

[10] Martin DD, Beauchamp E, Berthiaume LG. Post-translational myristoylation: Fat matters in cellular life and death. Biochimie. 2011;93:18–31. 10.1016/j.biochi.2010.10.018 21056615

[11] Dyda F, Klein DC, Hickman AB. GCN5-related N-acetyltransferases: A structural overview. Annu Rev Biophys Biomol Struct. 2000;29:81–103. 10.1146/annurev.biophys.29.1.81 10940244 PMC4782277

[12] Tillo SE, Xiong WH, Takahashi M, Miao S, Andrade AL, Fortin DA, et al Liberated PKA catalytic subunits associate with the membrane via myristoylation to preferentially phosphorylate membrane substrates. Cell Rep. 2017;19:617–629. 10.1016/j.celrep.2017.03.070 28423323 PMC5481286

[13] Arbuzova A, Schmitz AA, Vergeres G. Cross-talk unfolded: MARCKS proteins. Biochem J. 2002;362:1–12. 10.1042/0264-6021:3620001 11829734 PMC1222354

[14] Fong LWR, Yang DC, Chen C-H. Myristoylated alanine-rich C kinase substrate (MARCKS): A multirole signaling protein in cancers. Cancer Metastasis Rev. 2017;36:737–747. 10.1007/s10555-017-9709-6 29039083

[15] Moriya K, Yamamoto T, Takamitsu E, Matsunaga Y, Kimoto M, Fukushige D, et al Protein N-myristoylation is required for cellular morphological changes induced by two formin family proteins, FMNL2 and FMNL3. Biosci Biotechnol Biochem. 2012;76:1201–1209. 10.1271/bbb.120069 22790947

[16] Wang Y, Arjonen A, Pouwels J, Ta H, Pausch P, Bange G, et al Formin-like 2 promotes β1-integrin trafficking and invasive motility downstream of PKCα. Dev Cell. 2015;34:475–483. 10.1016/j.devcel.2015.06.015 26256210

[17] Kühn S, Erdmann C, Kage F, Block J, Schwenkmezger L, Steffen A, et al The structure of FMNL2-Cdc42 yields insights into the mechanism of lamellipodia and filopodia formation. Nat Commun. 2015;6:7088 10.1038/ncomms8088 25963737 PMC4432619

[18] Saito S, Hamamoto S, Moriya K, Matsuura A, Sato Y, Muto J, et al N-Myristoylation and *S*-acylation are common modifications of Ca^2+^-regulated *Arabidopsis* kinases and are required for activation of the SLAC1 anion channel. New Phytol. 2018;218:1504–1521. 10.1111/nph.15053 29498046

[19] Kinoshita E, Kinoshita-Kikuta E, Kubota Y, Takekawa M, Koike T. A Phos-tag SDS-PAGE method that effectively uses phosphoproteomic data for profiling the phosphorylation dynamics of MEK1. Proteomics. 2016;16:1825–1836. 10.1002/pmic.201500494 27169363

[20] Takamitsu E, Fukunaga K, Iio Y, Utsumi T. Cell-free identification of novel N-myristoylated proteins from complementary DNA resources using bioorthogonal myristic acid analogues. Anal Biochem. 2014;464:83–93. 10.1016/j.ab.2014.07.006 25043870

[21] Utsumi T, Matsuzaki K, Kiwado A, Tanikawa A, Kikkawa Y, Hosokawa T, et al Identification and characterization of protein N-myristoylation occurring on four human mitochondrial proteins, SAMM50, TOMM40, MIC19, and MIC25. PLoS One. 2018;13:e0206355 10.1371/journal.pone.0206355 30427857 PMC6235283

[22] Moriya K, Nagatoshi K, Noriyasu Y, Okamura T, Takamitsu E, Suzuki T, et al Protein N-myristoylation plays a critical role in the endoplasmic reticulum morphological change induced by overexpression of protein Lunapark, an integral membrane protein of the endoplasmic reticulum. PLoS One. 2013;8:e78235 10.1371/journal.pone.0078235 24223779 PMC3817238

[23] Kinoshita E, Kinoshita-Kikuta E. Improved Phos-tag SDS-PAGE under neutral pH conditions for advanced protein phosphorylation profiling. Proteomics. 2011;11:319–323. 10.1002/pmic.201000472 21204258

[24] Kinoshita-Kikuta E, Kinoshita E, Matsuda A, Koike T. Tips on improving the efficiency of electrotransfer of target proteins from Phos-tag SDS-PAGE gel. Proteomics. 2014;14: 2437–2442. 10.1002/pmic.201400380 25266391

[25] Kinoshita E, Kinoshita-Kikuta E, Takiyama K, Koike T. Phosphate-binding tag, a new tool to visualize phosphorylated proteins. Mol Cell Proteomics. 2006;5:749–757. 10.1074/mcp.T500024-MCP200 16340016

[26] Kinoshita E, Kinoshita-Kikuta E, Matsubara M, Yamada S, et al Separation of phosphoprotein isotypes having the same number of phosphate groups using phosphate-affinity SDS-PAGE. Proteomics. 2008;8:2994–3003. 10.1002/pmic.200800243 18615432

[27] Kinoshita E, Kinoshita-Kikuta E, Koike T. Separation and detection of large phosphoproteins using Phos-tag SDS-PAGE. Nat Protoc. 2009;4:1513–1521. 10.1038/nprot.2009.154 19798084

[28] Campellone KG, Welch MD. A nucleator arms race: Cellular control of actin assembly. Nat Rev Mol Cell Biol. 2010;11:237–251. 10.1038/nrm2867 20237478 PMC2929822

[29] Schonichen A, Geyer M. Fifteen formins for an actin filament: A molecular view on the regulation of human formins. Biochim Biophys Acta. 2010;1803:152–163. 10.1016/j.bbamcr.2010.01.014 20102729

[30] Goode BL, Eck MJ. Mechanism and function of formins in the control of actin assembly. Annu Rev Biochem. 2007;76:593–627. 10.1146/annurev.biochem.75.103004.142647 17373907

[31] Wallar BJ, Alberts AS. The formins: Active scaffolds that remodel the cytoskeleton. Trends Cell Biol. 2003;13:435–446. 10.1016/s0962-8924(03)00153-3 12888296

[32] Watanabe N, Higashida C. Formins: Processive cappers of growing actin filaments. Exp Cell Res. 2004;301:16–22. 15501440 10.1016/j.yexcr.2004.08.020

[33] Watanabe N, Madaule P, Reid T, Ishizaki T, Watanabe G, Kakizuka A, et al p140mDia, a mammalian homolog of Drosophila diaphanous, is a target protein for Rho small GTPase and is a ligand for profilin. EMBO J. 1997;16:3044–3056. 10.1093/emboj/16.11.3044 9214622 PMC1169923

[34] Faix J, Grosse R. Staying in shape with formins. Dev Cell. 2006;10:693–706. 10.1016/j.devcel.2006.05.001 16740473

[35] Suzuki T, Moriya K, Nagatoshi K, Ota Y, Ezure T, Ando E, et al Strategy for comprehensive identification of human N-myristoylated proteins using an insect cell-free protein synthesis system. Proteomics. 2010;10:1780–1793. 10.1002/pmic.200900783 20213681

[36] Li Y, Zhu X, Zeng Y, Wang J, Zhang X, Ding YQ, et al FMNL2 enhances invasion of colorectal carcinoma by inducing epithelial-mesenchymal transition. Mol Cancer Res 2010;8:1579–1590. 10.1158/1541-7786.MCR-10-0081 21071512

[37] Liang L, Li X, Zhang X, Lv Z, He G, Zhao W, et al MicroRNA-137, an HMGA1 target, suppresses colorectal cancer cell invasion and metastasis in mice by directly targeting FMNL2. Gastroenterology. 2013;144:624–635.e4. 10.1053/j.gastro.2012.11.033 23201162

[38] Zhu X-L, Zeng Y-F, Guan J, Li Y-F, Deng Y-J, Bian X-W, et al FMNL2 is a positive regulator of cell motility and metastasis in colorectal carcinoma. J Pathol. 2011;224:377–388. 10.1002/path.2871 21506128

[39] Utsumi T, Yoshinaga K, Koga D, Ide A, Nobori K, Okimasu E, et al Association of myristoylated protein with biological membrane and its increased phosphorylation by protein kinase C. FEBS Lett. 1988;238:13–16. 10.1016/0014-5793(88)80215-1 3169245

[40] Taniguchi H. Protein myristoylation in protein–lipid and protein–protein interactions. Biophys Chem. 1999;82:129–137. 10.1016/s0301-4622(99)00112-x 10631796

[41] Nishikawa K, Toker A, Johannes F-J, Songyang Z, Cantley LC. Determination of the specific substrate sequence motifs of protein kinase C isozymes. J Biol Chem. 1997;272:952–960. 10.1074/jbc.272.2.952 8995387

[42] Olsen JV, Blagoev B, Gnad F, Macek B, Kumar C, Mortensen P, et al Global, in vivo, and site-specific phosphorylation dynamics in signaling networks. Cell. 2006;127:635–648. 10.1016/j.cell.2006.09.026 17081983

