# Supplementary material for: A strategy to identify protein-N-myristoylation-dependent phosphorylation reactions of cellular proteins by using Phos-tag SDS-PAGE
Source: PLoS One. 2019 Nov 21;14(11):e0225510. doi: 10.1371/journal.pone.0225510 (PMC6872159; doi:10.1371/journal.pone.0225510)
Supplement: S1 Table — (DOCX) [file pone.0225510.s001.docx]

**S1 Table.** **PCR primers for FMNL2 mutagenesis.**

| **Primer** | **Sequence (5'–3')** |
| --- | --- |
| T92A_F | CTGGATCCAGCTGTAGCCAGGAAGAAATTCAGA |
| T92A _R | TCTGAATTTCTTCCTGGCTACAGCTGGATCCAG |
| S171A _ F | AAGCCCTGGAGTAGGGCCATCGAGGACCTGCAC |
| S171A _R | GTGCAGGTCCTCGATGGCCCTACTCCAGGGCTT |
| S179A _F | GACCTGCACAGAGGGGCCAACCTGCCCTCACCT |
| S179A _R | AGGTGAGGGCAGGTTGGCCCCTCTGTGCAGGTC |
| S183A _F | GGGAGCAACCTGCCCGCACCTGTGGGCAACAGT |
| S183A _R | ACTGTTGCCCACAGGTGCGGGCAGGTTGCTCCC |
| S188A _F | TCACCTGTGGGCAACGCTGTCTCCCGCTCTGGA |
| S188A _R | TCCAGAGCGGGAGACAGCGTTGCCCACAGGTGA |
| Y200A _F | CATTCTGCACTGCGAGCTAATACATTGCCAAGC |
| Y200A _R | GCTTGGCAATGTATTAGCTCGCAGTGCAGAATG |
| T202A _F | GCACTGCGATATAATGCATTGCCAAGCAGAAGA |
| T202A _R | TCTTCTGCTTGGCAATGCATTATATCGCAGTGC |
| Y234A _F | CGTGCCATCATGAATGCTCAGTATGGTTTCAAC |
| Y234A _R | GTTGAAACCATACTGAGCATTCATGATGGCACG |
| Y236A _F | ATCATGAATTATCAGGCTGGTTTCAACATGGTC |
| Y236A _R | GACCATGTTGAAACCAGCCTGATAATTCATGAT |
| S403A _F | CTGGAAGAAAACATTGCTCATTTATCTGAAAAA |
| S403A _R | TTTTTCAGATAAATGAGCAATGTTTTCTTCCAG |
| S406A _F | AACATTTCTCATTTAGCTGAAAAACTGCAAGAC |
| S406A _R | GTCTTGCAGTTTTTCAGCTAAATGAGAAATGTT |
| T412A _F | GAAAAACTGCAAGACGCAGAGAATGAAGCCATG |
| T412A _R | CATGGCTTCATTCTCTGCGTCTTGCAGTTTTTC |
| S418A _F | GAGAATGAAGCCATGGCCAAGATTGTGGAACTG |
| S418A _R | CAGTTCCACAATCTTGGCCATGGCTTCATTCTC |
| Y441A _F | GTCGTTCGGGAAATCGCCAAAGATGCAAATACT |
| Y441A _R | AGTATTTGCATCTTTGGCGATTTCCCGAACGAC |
| T450A _F | AATACTCAAGTTCACGCATTAAGAAAAATGGTC |
| T450A _R | GACCATTTTTCTTAATGCGTGAACTTGAGTATT |
| S466A _F | GCAATTCAAAGACAGGCTACCCTGGAAAAAAAG |
| S466A _R | CTTTTTTTCCAGGGTAGCCTGTCTTTGAATTGC |
| T467A _F | ATTCAAAGACAGTCTGCCCTGGAAAAAAAGATT |
| T467A _R | AATCTTTTTTTCCAGGGCAGACTGTCTTTGAAT |
| S522A _F | ACAATGGGGGCCGCTGCCTCAGGACCCTTGCCC |
| S522A _R | GGGCAAGGGTCCTGAGGCAGCGGCCCCCATTGT |
| S523A _F | ATGGGGGCCGCTTCCGCAGGACCCTTGCCCCCT |
| S523A _R | AGGGGGCAAGGGTCCTGCGGAAGCGGCCCCCAT |
| T799A _F | AGCATTCAGATGCTGGCTCCTCAACTACATGCG |
| T799A _R | CGCATGTAGTTGAGGAGCCAGCATCTGAATGCT |
| Y831A _F | TTAGCCCTTGGAAACGCCATGAATAGCAGTAAA |
| Y831A _R | TTTACTGCTATTCATGGCGTTTCCAAGGGCTAA |
| S834A _F | GGAAACTACATGAATGCCAGTAAAAGAGGAGCA |
| S834A _R | TGCTCCTCTTTTACTGGCATTCATGTAGTTTCC |
| S835A _F | AACTACATGAATAGCGCTAAAAGAGGAGCAGTT |
| S835A _R | AACTGCTCCTCTTTTAGCGCTATTCATGTAGTT |
| S1016A _F | CAGCAGGATCCAAAGGCTCCTTCTCATAAATCA |
| S1016A _R | TGATTTATGAGAAGGAGCCTTTGGATCCTGCTG |
| S1018A _F | GATCCAAAGTCTCCTGCTCATAAATCAAAGAGG |
| S1018A _R | CCTCTTTGATTTATGAGCAGGAGACTTTGGATC |
| S1072A _F | GATGCGGTGAGGAGAGCCGTCAGGCGGCGCTTT |
| S1072A _R | AAAGCGCCGCCTGACGGCTCTCCTCACCGCATC |
