# Supplementary material for: A strategy to identify protein-N-myristoylation-dependent phosphorylation reactions of cellular proteins by using Phos-tag SDS-PAGE
Source: PLoS One. 2019 Nov 21;14(11):e0225510. doi: 10.1371/journal.pone.0225510 (PMC6872159; doi:10.1371/journal.pone.0225510)
Supplement: S2 Table — (DOCX) [file pone.0225510.s002.docx]

**S2 Table.** **PCR primers for FMNL3 mutagenesis.**

| **Primer** | **Sequence (5'–3')** |
| --- | --- |
| S93A _F | AGCTTCTTGGACCCCGCTGTAACTCGGAAGAAG |
| S93A _R | CTTCTTCCGAGTTACAGCGGGGTCCAAGAAGCT |
| T95A _ F | TTGGACCCCAGTGTAGCTCGGAAGAAGTTCAGG |
| T95A _R | CCTGAACTTCTTCCGAGCTACACTGGGGTCCAA |
| S174A _F | CGGTCCTGGAGCAGGGCAATCGAGGACCTGCAG |
| S174A _R | CTGCAGGTCCTCGATTGCCCTGCTCCAGGACCG |
| S191A _F | GCCCCCTTCACCAACGCCCTCGCTCGCTCTGCG |
| S191A _R | CGCAGAGCGAGCGAGGGCGTTGGTGAAGGGGGC |
| T443A _F | GAGAGCATCAAGGAGGCATATGAGAACACAAGC |
| T443A _R | GCTTGTGTTCTCATATGCCTCCTTGATGCTCTC |
| Y444A _F | AGCATCAAGGAGACAGCTGAGAACACAAGCCAC |
| Y444A _R | GTGGCTTGTGTTCTCAGCTGTCTCCTTGATGCT |
| T447A _F | GAGACATATGAGAACGCAAGCCACCAGGTGCAC |
| T447A _R | GTGCACCTGGTGGCTTGCGTTCTCATATGTCTC |
| S448A _F | ACATATGAGAACACAGCCCACCAGGTGCACACC |
| S448A _R | GGTGTGCACCTGGTGGGCTGTGTTCTCATATGT |
| T687A _F | ATGCGCTTCCTGCCCGCAGAGGCTGAGGTAAAG |
| T687A _R | CTTTACCTCAGCCTCTGCGGGCAGGAAGCGCAT |
| Y697A _F | AAGCTGCTGCGGCAAGCTGAGCGGGAGCGGCAG |
| Y697A _R | CTGCCGCTCCCGCTCAGCTTGCCGCAGCAGCTT |
