# Supplementary figures and images for: A strategy to identify protein-N-myristoylation-dependent phosphorylation reactions of cellular proteins by using Phos-tag SDS-PAGE
Source: PLoS One. 2019 Nov 21;14(11):e0225510. doi: 10.1371/journal.pone.0225510 (PMC6872159; doi:10.1371/journal.pone.0225510)

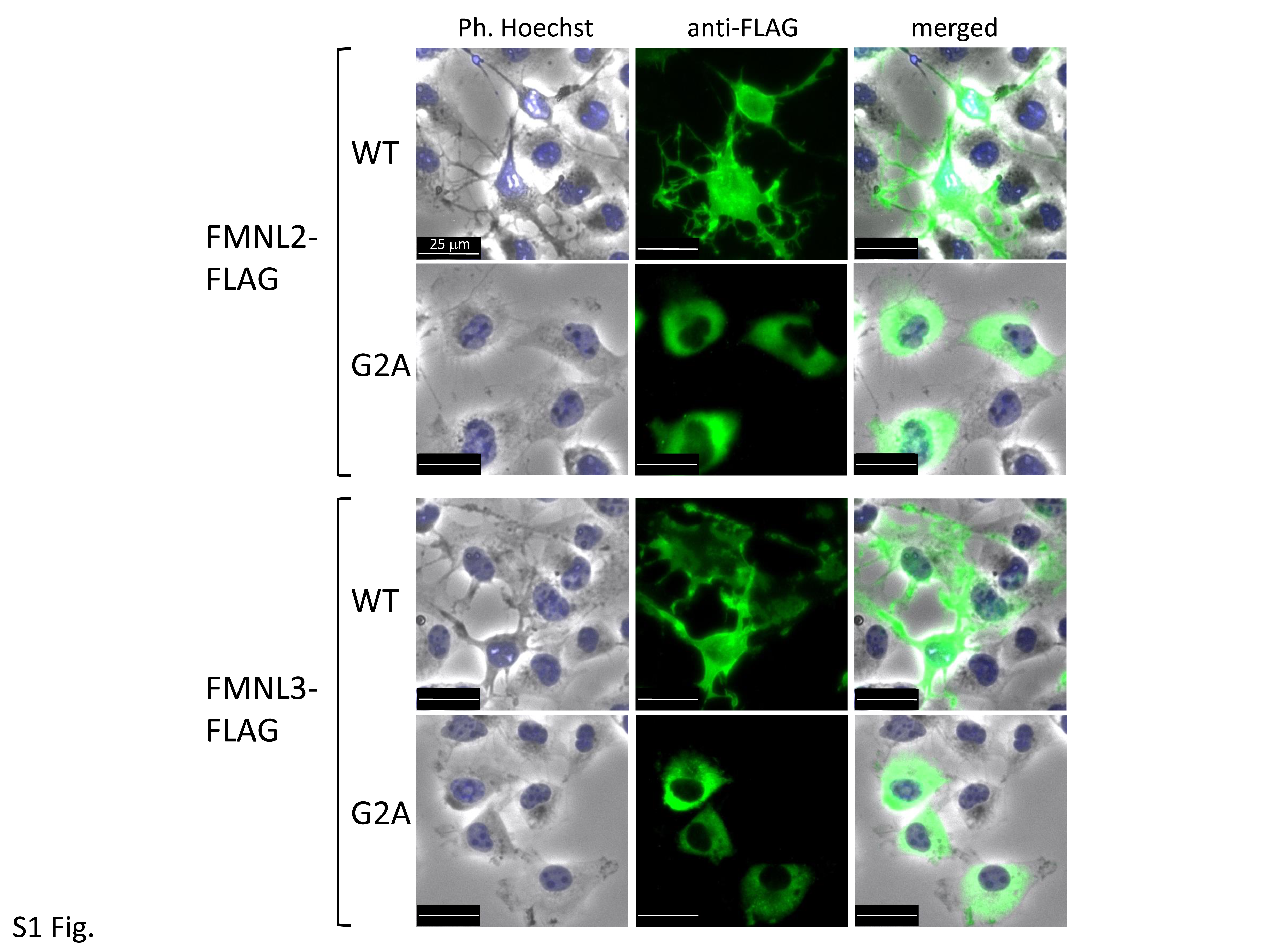

Supplement: S1 Fig — The merged images of phase contrast images and fluorescence microscopic images of COS-1 cells transfected with cDNA coding for wild type and G2A-mutants of FMNL2-FLAG and FMNL3-FLAG presented in Fig 2 were shown to demonstrate that induction of cellular morphological changes by FMNL2 and FMNL3 is dependent on protein N-myristoylation. (TIF) [file pone.0225510.s003.tif]
